# Olfactory receptor neurons are sensitive to stimulus onset asynchrony: implications for odor source discrimination

**DOI:** 10.1093/chemse/bjae030

**Published:** 2024-08-12

**Authors:** Georg Raiser, C Giovanni Galizia, Paul Szyszka

**Affiliations:** Department of Neurobiology, University Konstanz, Konstanz, Germany; International Max-Planck Research School for Organismal Biology, Konstanz, Germany; Champalimaud Neuroscience Programme, Champalimaud Foundation, Lisbon, Portugal; Department of Neurobiology, University Konstanz, Konstanz, Germany; Department of Neurobiology, University Konstanz, Konstanz, Germany; Department of Zoology, University of Otago, Dunedin, New Zealand

**Keywords:** figure–ground segregation, odor source segregation, lateral inhibition, ephaptic coupling, neuronal compartmentalization, odor mixtures, turbulent diffusion

## Abstract

In insects, olfactory receptor neurons (ORNs) are localized in sensilla. Within a sensillum, different ORN types are typically co-localized and exhibit nonsynaptic reciprocal inhibition through ephaptic coupling. This inhibition is hypothesized to aid odor source discrimination in environments where odor molecules (odorants) are dispersed by wind, resulting in turbulent plumes. Under these conditions, odorants from a single source arrive at the ORNs synchronously, while those from separate sources arrive asynchronously. Ephaptic inhibition is expected to be weaker for asynchronous arriving odorants from separate sources, thereby enhancing their discrimination. Previous studies have focused on ephaptic inhibition of sustained ORN responses to constant odor stimuli. This begs the question of whether ephaptic inhibition also affects transient ORN responses and if this inhibition is modulated by the temporal arrival patterns of different odorants. To address this, we recorded co-localized ORNs in the fruit fly *Drosophila melanogaster* and exposed them to dynamic odorant mixtures. We found reciprocal inhibition, strongly suggesting the presence of ephaptic coupling. This reciprocal inhibition does indeed modulate transient ORN responses and is sensitive to the relative timing of odor stimuli. Notably, the strength of inhibition decreases as the synchrony and correlation between arriving odorants decrease. These results support the hypothesis that ephaptic inhibition aids odor source discrimination.

## Introduction

**Fig. 1.**
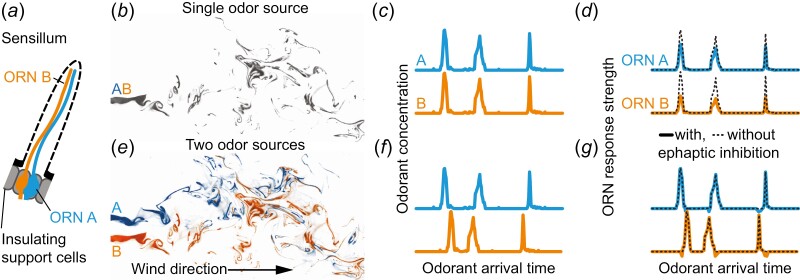
Hypothesis: Ephaptic inhibition is sensitive to relative stimulus timing and aids odor source discrimination. In a sensillum (*a*), co-localized ORNs inhibit each other’s response via ephaptic inhibition. Most natural odor stimuli are transient and fluctuate as a result of dispersion in turbulent plumes in the air. Different odorants (A and B) from a single source (*b*) arrive synchronously at olfactory receptor neurons (ORN) (*c*), making the odorant mixture’s neural representation different from the sum of its parts A and B (*d*). Odorants (A and B) from different sources (*e*) arrive asynchronously at the ORNs (*f*). Since ephaptic inhibition is active when the affected ORN exhibits weak or no response, it affects the neural responses less (*g*). This results in the neural representation of the odorant mixture resembling the sum of its individual components, A and B. Data in (*b*) and (*e*) from (Kree et al. 2013), data in (*c*), (*d*), (*f*), and (*g*) is fabricated.

Ephaptic coupling is a form of communication between neurons via direct electrical interaction that does not involve chemical or electrical synapses (Jefferys 1995). This mechanism requires close electrical proximity among neurons, achieved by sharing an electrically insulated extracellular space (Jefferys 1995). Such proximity is typical for insect olfactory receptor neurons (ORNs): ORNs are housed in sensilla (Fig. 1a), where multiple ORNs share the same extracellular space and are electrically insulated from the rest of the body (Redkozubov 1995). Electrical modeling has predicted that co-localized insect ORNs mutually inhibit each other through ephaptic coupling (Vermeulen and Rospars 2004), and such inhibition was experimentally demonstrated in fruit flies (Su et al. 2012; Zhang et al. 2019; Wu et al. 2022): When an ORN *A* is constantly stimulated with its cognate odorant A (which does not activate a co-localized ORN *B*), and ORN *B* is stimulated with a pulse of its cognate odorant B (which does not activate ORN *A*), then the sustained response of ORN *A* is inhibited by the response of ORN *B*.

It has been hypothesized that fast and temporally precise ephaptic inhibition between ORNs enables insects to detect whether different odorants originate from the same or from different sources (Fig. 1) (Baker et al. 1998). This hypothesis was motivated by the following observation in corn earworm moths (*Helicoverpa zea*) (Baker et al. 1998): Male moths take off for a search flight when they encounter packets of a female moth’s sex pheromone component (A) and also when A is partially mixed with sex pheromone packets (B) of another moth species. However, they do not take off when A and B are released from the same source. This behavioral inhibition has been explained by the synchrony between the arrival of A and B resulting from the common origin and complete mixing (Fig. 1b and c)—indicating that the source is a female moth from a different species. Conversely, if A and B arrive asynchronously, they likely originate from different sources, such as 2 female moths, 1 conspecific and the other from a different species (Fig. 1e and f). Ephaptic inhibition could enhance this discrimination between synchrony and asynchrony: In corn earworm moths, ORNs tuned to own and alien pheromone components are co-localized in a single sensillum (ORN A and ORN B in Fig. 1a). The mutual inhibition between ORN A and ORN B would become apparent when odorants A and B arrive synchronously (Fig. 1b–d), and the inhibited response of ORN A would then fail to trigger a search flight. A recent study modeled such an odor source discrimination mechanism (Pannunzi and Nowotny 2021).

For this source discrimination mechanism to work, we predict that in a turbulent stimulus situation, ephaptic inhibition would affect the transient ORNs’ responses to the onset of an odor stimulus, and the strength of synaptic inhibition would decrease with increasing asynchrony between arriving odorants. However, this has, to our knowledge, not yet been shown experimentally.

Here, we tested whether ephaptic inhibition affects transient ORN responses to odorant onset and to fluctuating odorant stimuli by recording a pair of ORNs within 1 fruit fly sensillum (the ab3A and ab3B ORNs). We chose the ab3 sensillum since it is known that ab3A and ab3B reciprocally inhibit each other’s sustained responses to constant odorant stimuli via ephaptic coupling (Su et al. 2012). Our results show that the strength of inhibition between those ORNs is modulated by the relative timing of odorant onsets, and thus could support odor source segregation in a natural environment: Mixtures of synchronously or correlated fluctuating odorant stimuli elicit inhibition, whereas mixtures of asynchronously or uncorrelated fluctuating odorant stimuli elicit weaker or no inhibition. The synchrony window of ORN’s inhibition is between 48 and 96 ms wide (range of onset asynchrony that induces inhibition), aligning with the 33 ms synchrony window observed in behavioral experiments (Sehdev et al. 2019).

## Materials and methods

### Single sensillum recordings in *Drosophila melanogaster*

**Fig. 2.**
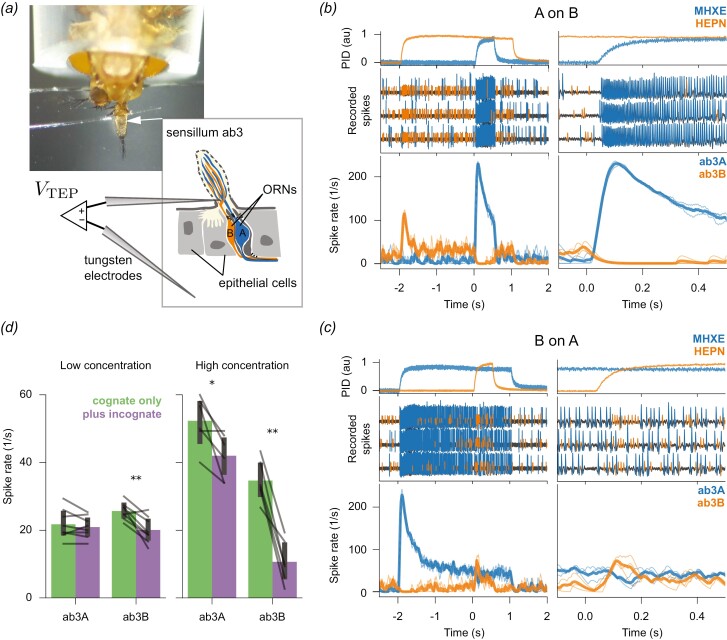
Confirmation of ephaptic inhibition of sustained ORN responses to constant odorant stimuli. *(a)* Fruit fly preparation for single sensillum recordings (SSR). We recorded from the ab3 sensillum which contains 2 olfactory receptor neurons: The larger ab3A neuron and the smaller ab3B neuron. Both neurons were simultaneously recorded with 1 tungsten electrode. *V*_TEP_, transepithelial potential (scheme of sensillum adapted from (Su et al. 2012)). (*b*) *Top*: Time course of the 2 odorant stimuli, methyl hexanoate (MHXE), and 2-heptanone (HEPN), measured with a photoionization detector (PID). MHXE is the cognate odorant for ab3A, and HEPN is the cognate odorant for ab3B. A pulse of MHXE was introduced into a constant background of HEPN, both at high concentrations. The moment the valve opened for the MHXE pulse is marked as Time = 0. The right panels provide a detailed view of the left. *Middle*: Recorded voltage traces of 3 individual trials from an ab3 sensillum during stimulation with high-concentration odorants. Spikes were automatically detected, with spikes from ab3A marked in blue and those from ab3B marked in orange. *Bottom*: Instantaneous spike rates of three ab3A (thin blue lines) and ab3B (thin orange lines) along with their means (bold lines). Note that color indicates odorant in the top traces, and their cognate neurons in the lower 2. (*c*) same as (*b*) but the 2 odorants were interchanged. (*d*) Spike rates, recorded during 0.1 to 0.35 s after opening of the valve for the cognate odorant, are shown without (green) and with the incognate odorant (purple). Data from 8 flies for low and 6 flies for high odorant concentrations, each stimulus was repeated 3 times. Bars show mean, and error bars show 95% confidence intervals. Stars show the significance level of paired *t*-tests (*P < 0.05; **P < 0.005). A color version of this figure appears in the online version of this article.

To measure the activity of multiple neurons within a single sensillum, we performed single sensillum recordings (Fig. 2a) (de Bruyne et al. 2001) and recorded from large basiconic ab3 sensilla of female *D. melanogaster* wild-type Canton S flies, aged 1 to 9 d. The flies were raised at 25 °C on a standard *D. melanogaster* medium, with a 12/12 h day/night cycle. Recordings were performed during the flies’ light period. Flies were immobilized in a pipette tip and mounted ventral side facing upwards on a custom 3D-printed holder. The antennae were bent outwards from the head and stabilized by a small droplet of n-eicosane (Sigma–Aldrich) onto the edge of a microscopy glass slide. Sensilla were localized using a 50× magnification air lens (50×/0.55 DIC EC Epiplan-Neofluar, Zeiss) with brightfield illumination. The recording and reference electrodes were tungsten wires (diameter = 0.1 mm), which were electrolytically sharpened with AC-current in a 0.5 M KOH solution. The recording electrode was inserted into the sensillum with a micromanipulator (Sensapex, SMXS-K-R). The reference electrode was inserted into the eye. The voltage difference between sensillar lymph and hemolymph (the transepithelial potential) was amplified 1,000× with an MA 103 head stage and MA 102 amplifier (Universität zu Köln) in DC mode, bandpass-filtered between 1 and 8,000 Hz. Noise from the powerline was reduced by a Hum Bug (Quest Scientific). Signals were digitized using a Micro 3 1401 A/D converter and recorded with the Spike 2 software (Cambridge Electronic Design). Sensillar identity was determined by applying a sequence of diagnostic odorants that unambiguously identify the sensillar identity (ethyl hexanoate, methyl acetate, isobutyl acetate, 2-butanone, 2,3-butanedione, and 1-hexanol, derived from the DoOR database (Münch and Galizia 2016)).

### Odorant stimuli

We used the odorants methyl hexanoate (MHXE, CAS: 106-70-7) and 2-heptanone (HEPN, CAS: 110-43-0) (Sigma–Aldrich). Odorant stimuli were generated with a custom-made stimulator optimized for temporal precision in binary mixtures (Raiser et al. 2016). We upgraded the published version of this stimulator with an air dilution system (Fig. S1a) which eliminated a decrease in odorant concentration within and across repeated stimulations (Fig. S1b). Odorants were diluted in air to 1.43 × 10^–3^ for “high concentration” or to 1.43 × 10^–6^ for “low concentration.” Throughout the experiment, vials containing the undiluted, pure odorant were continuously flushed with clean air to ensure a steady-state concentration of odorants in the headspace due to a balance between odorant evaporation and removal by the air flush. As a result of this continuous airflow, the headspace odorant concentration never reached saturation. Odorants were released at a rate of 3 mL/min and mixed with 297 mL/min of air (for the “low concentrations” this step was repeated 3 times). The 2 odorant channels were then combined and injected into a carrier air stream flowing at 1.5 L/min, resulting in a total outlet airflow from the stimulator of 2.1 L/min and a wind speed of 1.2 m/s.

To compare the actual stimulus concentrations used in our study with the odor dilutions reported by Su et al. (2012), one can examine the ORN spike rate. However, our spike rates during the odorant onset and constant stimulation likely overestimate the actual odor concentration.

#### Odorant onset.

**Fig. 3.**
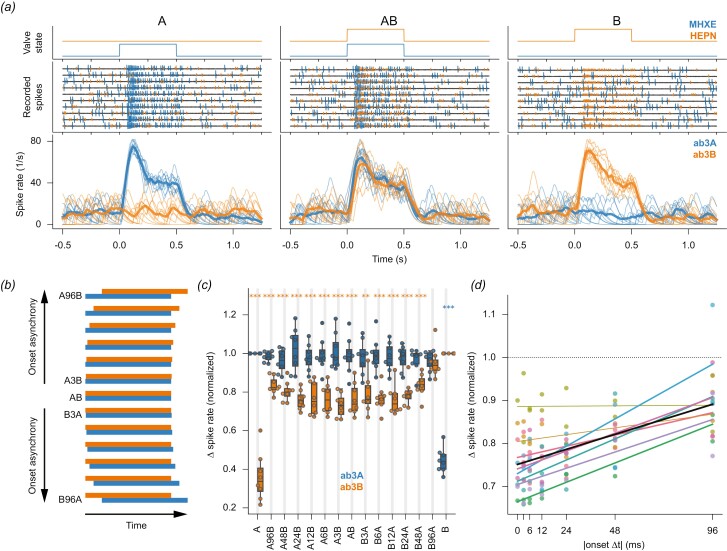
Relative onset timing in the millisecond range modulates ephaptic inhibition strength. (*a*) *Top*: Valve states for delivering 0.5-s long pulses of MHXE (blue) and HEPN (orange). *Middle*: Recordings from an ab3 sensilla in ten trials to low-concentrated odorants. Spikes from ab3A (blue) and ab3B (orange). *Bottom*: Instantaneous spike rates of ab3A (thin blue lines) and ab3B (thin orange lines) along with their means (bold lines). The moment the valve opened for the odorant pulses is marked as Time = 0.0. (*b*) True to scale temporal patterns of 0.5-s-long pulses of MHXE (blue, odorant A) and HEPN (orange, odorant B) in mixtures with varying stimulus onset asynchronies. (*c*) Transient responses, quantified as response peak rate, of ab3A (blue) and ab3B (orange) for all stimuli at low concentration across 7 flies. The spike rate is normalized to the cognate odorant response. Note the increase in inhibition, indicated by a lower firing rate, as onset time differences decrease towards the center. Circles show individual data points, horizontal lines show medians, boxes show interquartile ranges, and whiskers extend to ±1.5 times the interquartile ranges. Stars show significance levels for the comparison of each ORN’s response to the given stimulus and its response to the cognate stimulus alone, colored according to the tested ORN (two-tailed *t*-test, corrected for false discovery rate after (Verhoeven et al. 2005). *P < 0.05; **P < 0.005; ***P < 0.0005). (*d*) Same data as in (c), but only for ab3B, and pooling trailing and lagging onset time shifts (see Fig. S2 for the same analysis for ab3A). Data from 8 flies, each stimulus was presented 10 times. Each fly’s data is represented by a unique color. The colored lines show the linear regression for each fly (bold if *P* < 0.05), and the black line shows the average regression (*r*^2^ = 0.31, *P* < 0.0005). The positive slope shows that ephaptic inhibition increases with increasing synchrony between odorant onsets. A color version of this figure appears in the online version of this article.

Our spike rates were 70 to 75 spikes/s for “low concentrations” (Fig. 3a) and 120 to 230 spikes/s for “high concentrations” (Fig. 2b and c). These rates correspond to 70 to 200 spikes/s for concentrations between 10^–6^ and 10^–4^ in Su et al. (Fig. 3b). Note that the ORN spike rate increases with both odorant concentration and the speed of concentration rise. Our stimulator was designed to achieve maximum concentration rise speed, thus our higher spike rates may reflect a faster rise rather than a higher concentration.

#### Constant stimulation.

Our spike rates were 21 to 25 spikes/s for “low concentrations” (Fig. 3a) and 35 to 52 spikes/s for “high concentrations” (Fig. 2b and c). These rates correspond to 23 to 50 spikes/s for dilutions between 5 × 10^–8^ and 5 × 10^–7^ in Su et al. (Fig. 3d). We measured the sustained response just 2 s after odorant onset, while Su et al. used a permanent background odorant. Therefore, our spike rates during constant stimulation might be less attenuated by adaptation, implying our “low concentration” might be below those used by Su and colleagues (Su et al. 2012).

**Fig. 4.**
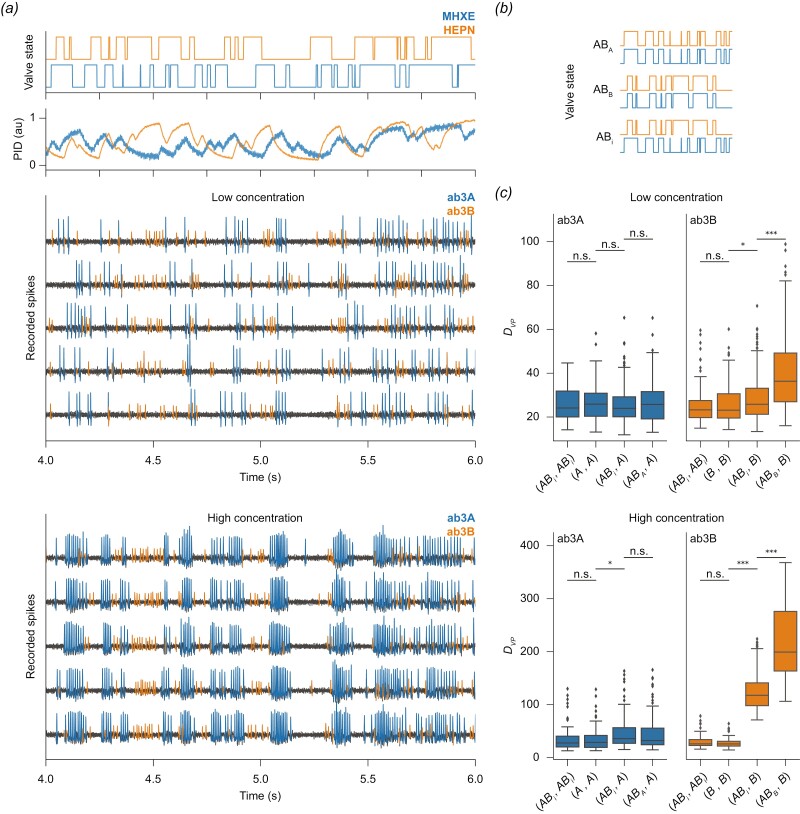
Ephaptic inhibition is stronger for correlated than for uncorrelated fluctuating odorant stimuli. (*a*) Valve states for delivering mixed, but uncorrelated fluctuating streams of odorant A (MHXE, blue) and B (HEPN, orange). Time course of the 2 odorant stimuli A and B measured with a photoionisation detector (PID). Recorded spikes from an ab3 sensillum in 5 different trials with automatically detected ab3A spikes (blue) and ab3B spikes (orange). (b) Valve states for delivering mixed, correlated fluctuating streams of odorant A and B (AB_A_ or AB_B_; the index indicates whether A and B fluctuated with the time course A or B) and for mixed, uncorrelated fluctuating odorant streams (AB_*i*_). (*c*) Victor–Purpura spike train distances (*D*_VP_) for different comparisons of correlated and uncorrelated odorant mixture stimulations. Box plots show the distribution of within-animal pairwise comparisons for different stimulus pairs of single odorants (A or B), correlated mixtures (AB_A_ or AB_B_), and uncorrelated mixtures (AB_*i*_). Low values indicate similar spike trains and higher values indicate different spike trains. The effects are strongest in high concentration, ab3B: Spike trains are highly reproducible (low *D*_VP_ values for (B, B)), and highly reproducible when an incognate odorant is added (low *D*_VP_ values for (AB_*i*_, AB_*i*_)). However, adding an incognate odorant changes the response patterns as compared to the cognate odorant alone [(AB_*i*_, B) and (AB_B_, B) as compared to (AB_*i*_, AB_*i*_) and (B, B)]. Ephaptic coupling increases when the 2 odorants are correlated [*D*_VP_ values are higher for (AB_B_, B) than for (AB_*i*_, B)]. The same results are found for ab3B at low concentration, but the effect is weaker for ab3A at high concentration and absent for ab3A at low concentration. Horizontal lines mark the medians, boxes show interquartile ranges, whiskers extend to ±1.5 times the interquartile ranges, and outliers are drawn as points. Stars show significance levels (two-sided *U* test; *P < 0.05; **P < 0.005; ***P < 0.0005). Data from 6 flies for the low concentration and 8 flies for the high concentration. Each stimulus was presented 5 times. A color version of this figure appears in the online version of this article.

Valves of the olfactory stimulator were controlled by a CompactRIO system equipped with digital I/O modules NI-9403 and by software written by Stefanie Neupert in LabVIEW 2011 SP1 (National Instruments). In our study, odorant stimuli were either brief pulses of odorants added into a longer-lasting background odorant (Fig. 2), pulses with varying onset delays in the millisecond range (Fig. 3), or randomly fluctuating odorant concentrations (Fig. 4). Reproducible streams of fluctuating odorant concentrations (Fig. 4a) were generated by switching the stimulator’s valves at pseudorandom time points, where the switching times were generated from a Poisson point process with mean interval of 50 ms for a 10 s long segment. Two sequences of valve state changes for both odorants A and B were calculated separately. From those sequences, different correlated and uncorrelated mixture stimuli were constructed: For the uncorrelated mixtures, each of the stimuli A and B followed its own independent random sequences (abbreviated AB_*i*_). For the correlated mixtures, both odorants A and B followed the identical time course, either of A (AB_A_) or of B (AB_B_) (see Fig. 4b for a graphical description).

### Data analysis

Spike sorting was performed with a spike sorting algorithm specifically tailored for single sensillum recordings, designed to capture the prominent change in spike shape as a function of firing rate (www.github.com/grg2rsr/sssort). All further data analysis was performed with custom Python scripts (www.github.com/grg2rsr/rgs_ephaptic).

Spike rate estimation was performed by convolving the estimated spike trains with an alpha kernel with a standard width of σ = 50 ms. An alpha kernel ensures that the predicted spike rates do not rise before an actual spike occurs (Nawrot et al. 1999).

Spike train dissimilarity was quantified as Victor–Purpura distance, *D*_VP_ (Victor and Purpura 1996). Briefly, *D*_VP_ between 2 spike trains is calculated by transforming one spike train into the other, and each transformation (removal of a spike, addition of a spike or shift by a defined time) is associated with a cost. The algorithm then finds the lowest cost to transform one spike train into the other. *D*_VP_ was computed with the function *spike_train_dissimilarity.victor_purpura_dist()* of the Python module *elephant* (version 0.12.0). Statistical testing for differences in *D*_VP_ was done by separate *U*-tests between the groups and a subsequent Bonferroni correction to adjust the *P*-values for multiple comparisons.

## Results

First, we aimed to confirm ephaptic inhibition between co-localized ORNs in ab3 sensilla, which contain 2 ORNs, ab3A, and ab3B (Fig. 2a) (de Bruyne et al. 2001). ab3A is larger than ab3B and can be recognized in single sensillum recordings by its larger spike amplitudes (Fig. 2b, c). We used the 2 odorants, methyl hexanoate (MHXE) and 2-heptanone (HEPN) (Su et al. 2012). MHXE activates ab3A (cognate odorant for ab3A) but not ab3B (incognate odorant for ab3B), while HEPN activates ab3B (cognate odorant for ab3B) but not ab3A (incognate odorant for ab3A).

Adding MHXE into a HEPN background led to a decreased HEPN response in ab3B (“A on B” in Fig. 2b). Conversely, adding HEPN into an MHXE background led to a decreased MHXE response in ab3A (“B on A” in Fig. 2c). These results corroborate the previously reported reciprocal but asymmetric ephaptic inhibition between the 2 neurons (Su et al. 2012). Next, we investigated the effect of odorant concentration (Fig. 2d) and confirmed that inhibition strength increases with odorant concentration (and, accordingly, response strength) (Su et al. 2012).

To test whether ab3A and ab3B inhibit each other’s transient response to odorant onset and whether relative onset timing between 2 odorants modulates ephaptic inhibition strength, we presented binary mixtures of MHXE and HEPN with shifted onsets between the 2 odorants (0, 3, 6, 12, 24, 48, or 96 ms onset asynchrony, with either of the 2 odorants as the trailing one) (Fig. 3a and b). This range covers the behaviorally relevant range, as behavioral experiments have demonstrated that fruit flies can detect odorant onset asynchronies as brief as 33 ms (Sehdev et al. 2019).

ORNs ab3A and ab3B gave reliable responses to these mixtures. However, the response of ab3B to the binary mixture was reduced as compared to the response to its ligand HEPN alone (Fig. 3c). The magnitude of this reduction increased with decreasing odorant-onset mismatch toward the center of the plot (Fig. 3d). Note that the lack of inhibition of ab3B’s response to odorant B during B96A stimulation might simply be due to ab3B reaching its response peak within 96 ms, before the arrival of odorant A. However, the reduced inhibition of ab3B’s response to odorant B during A96B stimulation cannot be explained by the absence of odorant A, as ab3B’s response to odorant B occurs in the presence of odorant A. The timing of ORNs’ response onsets was not systematically affected by ephaptic inhibition (Fig. S3).

In the experiments conducted so far, all odorant stimuli were square pulses, meaning there was a sudden increase of the odorant concentration from 0 to either low or high concentration. However, naturally occurring odorant stimuli exhibit complex temporal fluctuations in concentration. Importantly, odorants from spatially separated sources have uncorrelated temporal structures, while odorants (i.e. different chemicals in a blend) from a single source have correlated temporal structures (Fig. 1b and c) (Kree et al. 2013; Celani et al. 2014; Riffell et al. 2014; Soltys and Crimaldi 2015; Ackels et al. 2021). To investigate how stimulus correlation in a realistic environment influences ephaptic coupling, we compared ORN responses to mixtures where the streams of odorants A (MHXE) and B (HEPN) were either uncorrelated and independent (AB_*i*_) or completely correlated and synchronous (AB_A_ or AB_B_; the index indicates whether the shared time course of A and B corresponds to the component A or B in the AB_*i*_ stimulus) (depicted in Fig. 4b). Hence, AB_A_ and AB_B_ are 2 temporally different stimuli that share the same degree of temporal correlation, and AB_*i*_ is a stimulus in which the individual components share no temporal correlation, and all stimuli share the same amount of overall odorant concentrations.

To measure spike sequence similarity, we computed Victor–Purpura distances (*D*_VP_) (Victor and Purpura 1996) between all pairwise combinations of spike trains for the individual stimuli (Fig. 4c). Fluctuating odorant stimuli elicited reproducible spike sequences in both neurons (Fig. 4a), as quantified by low *D*_VP_ values when comparing repeated responses to the same stimuli [(AB_*i*_, AB_*i*_), (A, A), or (B, B)], giving a baseline level of *D*_VP_ resulting from the trial-to-trial variability.

In the absence of ephaptic interactions, adding an incognate odorant to a stimulus should have no effect, and thus responses of the ab3A neuron to the stimuli A, AB_*i*_, and AB_A_ should be equal, i.e. the *D*_VP_ for (A, A) should not differ from (AB_*i*_, A). Likewise, *D*_VP_ for responses of the ab3B neuron should be equal when comparing (B, B) to (AB_*i*_, B). However, we found that adding an incognate odorant led to significantly different responses: In the ab3B neuron, *D*_VP_ was greater for (AB_*i*_, B) than for (B, B) (*P* < 0.05 for low and *P* < 0.0005 for high concentrations), and in the ab3A neuron, *D*_VP_ was greater for (AB_*i*_, A) than for (A, A) (*P* < 0.05 for high concentrations). This shows that adding an incognate odorant in a turbulent stimulus led to ephaptic interaction.

Because the strength of ephaptic inhibition increased with increasing synchrony between stimulus onsets (Fig. 3), we hypothesize that the ephaptic interaction increases with increasing correlation of the time course of the stochastically fluctuating odorant stimuli. If this hypothesis holds true, then *D*_VP_ should be greater when the time courses of the incognate and cognate odorant are identical. In the ab3B neuron, this was indeed the case, as *D*_VP_ was larger for (AB_B_, B) than for (AB_*i*_, B) (*P* < 0.0005 for both tested concentrations).

This supports the hypothesis that ephaptic inhibition is sensitive to the correlation between odor streams, probably due to increased opportunities for ephaptic coupling (Fig. 1g). The absence of this effect in ab3A could be attributed to its larger size in comparison to ab3B: The smaller “B” neurons exert weaker ephaptic inhibition, and the larger “A” neurons are less susceptible to ephaptic inhibition (Zhang et al. 2019). As a result, the ephaptic inhibition from ab3B on ab3A might be too weak to be detected.

## Discussion

We tested Baker and colleagues’ hypothesis (Baker et al. 1998) that ephaptic inhibition between co-localized olfactory receptor neurons (ORNs) decreases with decreasing stimulus onset synchrony or correlation between fluctuating odorant streams, a mechanism that aids source discrimination (Fig. 1). To this end, we first confirmed previous studies which demonstrated that in the ab3 sensillum of *D. melanogaster* the response of the ORN ab3A to its cognate odorant MXHE inhibits a sustained responses of ab3B to a constant odorant stimulus of HEPN, and vice versa (Fig. 2) (Su et al. 2012; Zhang et al. 2019). Su and colleagues observed stronger ephaptic inhibition compared to our results. This is likely due to the relatively higher concentration of their incognate odor, which induces stronger inhibition of the cognate odor response. Specifically, Su and colleagues presented the ab3A-inhibiting incognate odorant at a concentration 1,000 times higher than the cognate odorant (10^–4^ vs 10^–7^dilution), and the ab3B-inhibiting incognate odorant at a concentration 5 times higher (10^–6^ vs 5 × 10^−7^ dilution). In contrast, we presented both odorants at the same dilution.

Unlike Su and colleagues, we did not conduct a control experiment to prove that this nonsynaptic inhibition reflects ephaptic coupling (e.g. by ablating one of the two ab3 neurons). However, given the strong correspondence between our findings and those of Su and colleagues, we conclude that the lateral inhibition that we found is also due to ephaptic inhibition.

Our key finding is that ephaptic inhibition also affects transient ORN responses when odorant onsets are synchronous (Fig. 3) and when odorant stimuli fluctuate (Fig. 4). This expands upon previous observations of ephaptic inhibition affecting sustained ORN responses to constant odorant stimuli (Su et al. 2012; Zhang et al. 2019; Wu et al. 2022). The systematic relationship between stimulus onset asynchrony and inhibition strength (Fig. 3c) can be explained as follows: Only with synchronous stimulus onsets do the peak responses coincide, allowing ab3A’s peak response to maximally inhibit ab3B’s peak response. With asynchronous stimulus onsets, ab3B’s peak response occurs either before ab3A reaches its peak (BΔ*t*A) or after ab3A’s response amplitude has already decayed (AΔ*t*B).

The finding that ephaptic inhibition is sensitive to stimulus onset asynchrony is relevant because constant and square odorant stimuli are rarely found in natural environments, where odorants fluctuate rapidly (Murlis et al. 1992; Celani et al. 2014). Furthermore, neural responses to odorant onset flanks were found to be more informative about the identity and concentration of an odorant than sustained responses (Vickers et al. 2001; Fernandez et al. 2009; Krofczik et al. 2009; Raman et al. 2010; Strube-Bloss et al. 2012; Jeanne and Wilson 2015; Kim et al. 2015; Egea-Weiss et al. 2018), and during odor-guided navigation, insects use the timing of odorant onsets to locate odor sources (Kennedy and Marsh 1974; Baker and Haynes 1989; Kanzaki et al. 1992; Mafra-Neto and Cardé 1994; Álvarez-Salvado et al. 2018; Pang et al. 2018; Demir et al. 2020; Jayaram et al. 2022, 2023; Szyszka et al. 2023). Therefore, our finding of ephaptic inhibition of transient ORN responses to odorant onsets suggests that this lateral inhibition plays a role in extracting information about odor identity, concentration, and odor source location. This emphasizes the significance of neuronal compartmentalization and raises the question about the evolutionary pressures that led to the association of specific ORNs into discrete sensilla (Ng et al. 2020; Pannunzi and Nowotny 2021; Wu et al. 2022).

Importantly, the strength of ephaptic inhibition between ORNs varied with relative odorant onset timing (Fig. 3c), as predicted by (Baker et al. 1998): Ephaptic inhibition was weakest with asynchronous and uncorrelated mixtures. In a natural setting, this would occur when two separate odorant sources create overlapping but desynchronized odorant plumes downwind (Fig. 1e–g), for example, when a female moth releases sex pheromone, and a sympatric female from another species releases her (different) pheromone from nearby (Baker et al. 1998). The reduction of ephaptic coupling in this scenario allows for a more efficient signal separation by the male moth, helping the male to identify its conspecific female.

In addition to discriminating between the synchronous and asynchronous arrival of different pheromones, the temporal sensitivity of ephaptic inhibition could aid general odor source discrimination. Odorants from a single source arrive at the antenna synchronously and as correlated odorant streams (Celani et al. 2014; Ackels et al. 2021). Since those single-source odorants induce ephaptic inhibition, they lead to distinct ORN responses compared to the single odorants. This facilitates synthetic odor processing, such that the odorants are perceived as one unitary object (a perfume or gestalt) and thus interpreted as coming from a single source. However, odorants from two different sources arrive at the antenna asynchronously and as uncorrelated odorant streams (Fig. 1) (Kree et al. 2013; Celani et al. 2014; Riffell et al. 2014; Soltys and Crimaldi 2015; Ackels et al. 2021). Those separate-source odorants induce less ephaptic inhibition, leading to similar ORN responses compared to the single odorants. This facilitates analytical odor processing, such that the odorants are perceived as a mixture of 2 distinct and recognizable compounds—or as coming from 2 separate sources.

The synchrony window triggering ephaptic inhibition in ORNs (the 48 to 96 ms odorant onset asynchrony, Fig. 3c) is consistent with behavioral experiments showing that fruit flies discriminate odor sources based on odorant onset asynchrony of 33 ms (Sehdev et al. 2019). This strengthens the hypothesis that ephaptic inhibition aids odor source discrimination based on the asynchronous onset of stimuli. This hypothesis is further supported by studies showing co-localization of ORNs that respond to odorants with opposing valences (Kaissling et al. 1978; Hansson et al. 1987; Nikonov and Leal 2002; Andersson et al. 2010; Ng et al. 2020; Wu et al. 2022), and studies showing behavioral responses to attractive odorants diminish with synchronous, but not asynchronous, arrival of antagonistic odorants (Nikonov and Leal 2002; Andersson et al. 2011; Szyszka et al. 2012; Weissburg et al. 2012; Saha et al. 2013; Sehdev et al. 2019). Exploring the temporal sensitivity of ephaptic inhibition between ORNs in the same insect species as in these studies will provide further insights into its functional role in odor source discrimination.

## Supplementary material

Supplementary material can be found at http://www.chemse.oxfordjournals.org/

bjae030_suppl_Supplementary_Material

## Data Availability

Data are archived at https://datadryad.org/stash/share/ivM6cAG_kwVSUEFnTlAmIl7Blejpq9579flj-24Bl-k. Code for spike sorting is available at https://github.com/grg2rsr/sssort and code for data analyzes is available at https://github.com/grg2rsr/rgs_ephaptic.
