# Supplementary material for: Olfactory receptor neurons are sensitive to stimulus onset asynchrony: implications for odor source discrimination
Source: Chem Senses. 2024 Aug 12;49:bjae030. doi: 10.1093/chemse/bjae030 (PMC11408607; doi:10.1093/chemse/bjae030)
Supplement: bjae030_suppl_Supplementary_Material [file bjae030_suppl_supplementary_material.docx]

Supplementary Materials

| 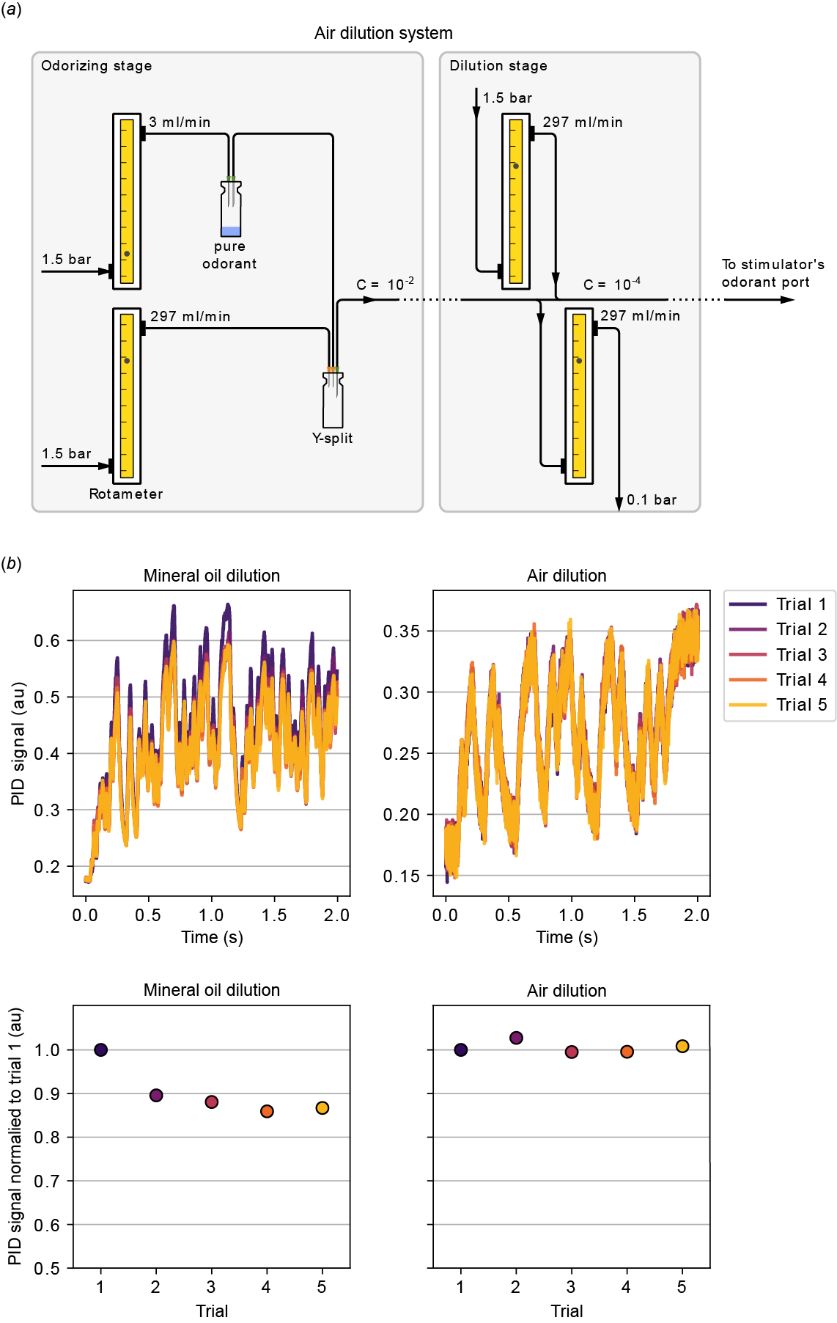 |
| --- |
| **Figure S1.** Air dilution system for stable odorant concentrations. (*a*) Pressure-controlled (35898; Analyt-MTC), charcoal-filtered air from in-house air supply was divided in a 1:100 ratio (3 and 297 ml/min) with flowmeters (112-02GL, Analyt-MTC). After the smaller fraction was routed through the headspace of a 20 ml glass vials (Schmidlin, sealed with a Teflon septum), both flows were combined. This served as an initial 10^-2^ dilution. Further dilutions were generated by first removing 297 ml/min, and subsequently adding 297 ml/min clean air to the air stream, thus diluting by another factor of 100. (*b*) *Top*: Raw PID signal to 5 consecutive fluctuating 2-heptanone stimuli, produced using a mineral oil dilution (*left*) and an air dilution (*right*) show the higher reproducibility of the air dilution system. The strength of the PID signal is proportional to odorant concentration. *Bottom*: Mean PID signal during 0.5-2.0 s, divided by the first trials’ value. In the mineral oil dilution system, the odorant concentration decreases from trial to trial, but in the air dilution system, the odorant concentration remains stable. |

| **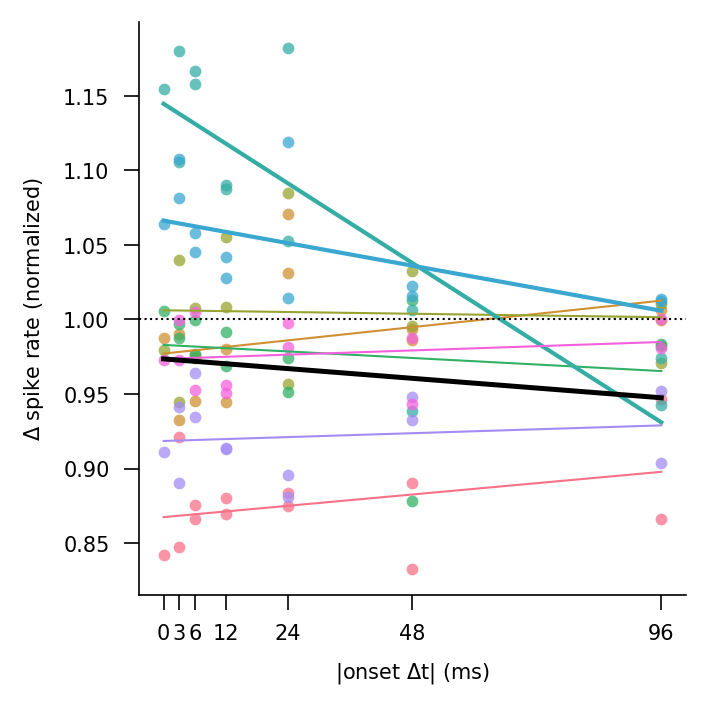** |
| --- |
| **Figure S2.** No evidence for ephaptic inhibition of the transient response peak rate of ab3A. Same data as in Fig 3d, but for ab3A. |

| **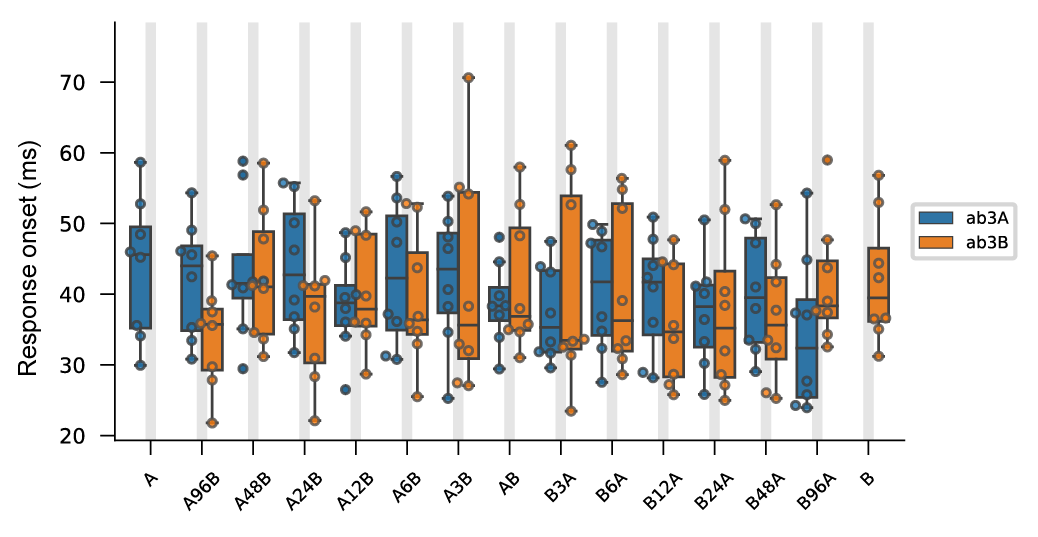** |
| --- |
| **Figure S3.** Ephaptic inhibition does not affect the timing of ORN’s odor response. Same data as in Fig. 3c, but the stimulus delay-corrected response peak timing was analysed. N = 7 sensilla in 7 flies. Horizontal lines show medians, boxes show interquartile ranges, and whiskers extend to ±1.5 times the interquartile ranges. |
